# Gliomas

**DOI:** 10.1155/2014/470523

**Published:** 2014-08-26

**Authors:** Giuseppe Lombardi, Alessandro Della Puppa, Anna Luisa Di Stefano, Andrea Pace, Roberta Rudà, Emeline Tabouret, Vittorina Zagonel

**Affiliations:** ^1^Medical Oncology 1, Veneto Institute of Oncology-IRCCS, Via Gattamelata 64, 35128 Padua, Italy; ^2^Neurosurgery Department, Padua Hospital, 35128 Padua, Italy; ^3^Department of Neuro-Oncology, UPMC, GH Pitié-Salpétrière, 75005 Paris, France; ^4^DBBS, National Neurological Institute C. Mondino, University of Pavia, 27100 Pavia, Italy; ^5^Neurology Unit, National Cancer Unit, Regina Elena, 00144 Rome, Italy; ^6^Department of Neuro-Oncology, Città della Salute e della Scienza, University of Turin, 10126 Turin, Italy; ^7^Department of Neuro-Oncology, Hopitaux de Marseille, Marseille, France

Gliomas are a heterogeneous group of tumors developing from glial cells in the central nervous system. Gliomas are divided into two histopathological subgroups: low and high grade gliomas. High-grade gliomas, such as glioblastoma and anaplastic astrocytoma, are extremely aggressive lesions and represent the most common primary malignant brain tumors.

In the last years, there have been important developments about their biologic mechanism, their surgical and drug treatment, and their diagnosis and genetic mutations; indeed, the recent IDH gene mutation identification in gliomas has been an important contribution to the knowledge improvement of biological mechanism and prognosis of these tumors. Through the analysis of IDH gene mutation it is possible to add molecular characteristics to refine the WHO classification in order to define more homogeneous gliomas subgroups. X.-W. Wang et al. showed that IDH mutation is almost constant in 1p19q codeleted tumors and they stratified low- and high-grade gliomas according to the codeletion of 1p19q and IDH mutation to define three prognostic subgroups: 1p19q and IDH mutated, IDH mutated alone, and none of these alterations; they demonstrated that the presence of IDH mutation combined with other genomic markers can be used to refine the prognostic classification of gliomas, independently of tumor grade. Noteworthy, X.-W. Wang et al., in another work, showed that IDH1-R132H mutation could be predictive of response to radiation therapy; indeed, they suggested that IDH mutation could increase radiosensitivity in hypoxic conditions, underlining the primordial IDH mutation determination whatever the diagnostic approach. Indeed, in a recent work, G. Lombardi et al. [1] reported the possibility to discriminate IDH mutation analyzing the concentration of 2-hydroxyglutarate in urinary and plasma samples.

As described by P. Gonzalez-Gomez et al., another signaling pathway such as bone morphogenetic proteins (BMPs) could present with both prognostic value and promising therapeutic tools for gliomas.

A very interesting study about the use of 5-aminolevulinic acid (5-ALA) fluorescence in high-grade gliomas surgery was reported by A. Della Puppa et al.; they analyzed 94 patients who underwent surgery guided by 5-ALA fluorescence and stratified data for recurrent surgery, tumor location, tumor size, and tumor grade; they concluded that this surgical approach enables a gross total resection in 100% of cases and recurrent surgery, location, size, and tumor grade can be predictor of surgical outcome. The role of salvage radiosurgery in patients with recurrent malignant gliomas was studied by M. Martínez-Carrillo et al.; retrospectively, they analyzed 87 patients with recurrent anaplastic astrocytoma and glioblastoma who underwent stereotactic radiosurgery; although the population was very heterogeneous and various prior studies showed conflicting results about the efficacy of reirradiation, they concluded that this treatment was safe and may be a potential treatment option in selected patients.

New technological instruments such as brain magnetic resonance imaging (MRI) with spectroscopy and perfusion can help in the right diagnosis for these tumors; in fact, A. L. Di Stefano et al., evaluating perfusion MRI in grades III and IV gliomas, showed that any significant difference in rCBV between grade III and grade IV is detectable in the contrast-enhancement area while areas of high perfusion on CBV map appear capable of best characterizing the degree of neovascularization and should be considered as the reference areas to be targeted for gliomas grading. The role of diffusion tensor histogram analysis was studied in pediatric diffuse intrinsic pontine gliomas by E. A. Steffen-Smith et al. from National Institutes of Health in Bethesda; they evaluated tumor structure in children using histogram analyses of mean diffusivity, concluding that this method can show significant interpatient and intratumoral differences and quantifiable changes in tumor structure.

Finally, an Italian study by V. Vaccaro et al. analyzed the efficacy of bevacizumab in association with fotemustine in patients with recurrent malignant gliomas. Antiangiogenic treatments for glioma patients have been tested in numerous clinical trials, both retrospective and prospective studies, with conflicting results; indeed, recently, two randomized prospective phase III studies failed to demonstrate the bevacizumab efficacy when added to temozolomide and radiation therapy for new glioblastoma patients [2, 3]. The combination treatment with bevacizumab and fotemustine was previously studied by R. Soffietti et al. [4] in recurrent glioblastoma patients, although with a different dosage and schedule. In both studies, this regimen showed interesting results with good safety in these patients.

In conclusion, gliomas represent an important subject of study and in this special issue very interesting works on recent developments about diagnosis, molecular biology, surgical treatment, and new targeted therapies for gliomas were selected.

*Giuseppe  Lombardi* *Alessandro  Della  Puppa* *Anna  Luisa  Di  Stefano* *Andrea  Pace* *Roberta  Rudà* *Emeline  Tabouret* *Vittorina  Zagonel*
